# Antibacterial potential associated with drug-delivery built TiO_2_ nanotubes in biomedical implants

**DOI:** 10.1186/s13568-019-0777-6

**Published:** 2019-04-16

**Authors:** Marcel Ferreira Kunrath, Bruna Ferreira Leal, Roberto Hubler, Sílvia Dias de Oliveira, Eduardo Rolim Teixeira

**Affiliations:** 1 Dentistry University, School of Health Sciences, Pontifical Catholic University of Rio Grande do Sul (PUCRS), Av. Ipiranga, P.O. Box 6681, Porto Alegre, 90619-900 Brazil; 2 Immunology and Microbiology Laboratory, School of Sciences, Pontifical Catholic University of Rio Grande do Sul (PUCRS), Av. Ipiranga, P.O. Box 6681, Porto Alegre, 90619-900 Brazil; 3 Materials and Nanoscience Laboratory, Pontifical Catholic University of Rio Grande do Sul (PUCRS), P.O. Box 1429, Porto Alegre, 90619-900 Brazil

**Keywords:** Antibacterial surfaces, Biomedical implants, Drug delivery, Nanoparticles, Surfaces, TiO_2_ nanotubes

## Abstract

The fast evolution of surface treatments for biomedical implants and the concern with their contact with cells and microorganisms at early phases of bone healing has boosted the development of surface topographies presenting drug delivery potential for, among other features, bacterial growth inhibition without impairing cell adhesion. A diverse set of metal ions and nanoparticles (NPs) present antibacterial properties of their own, which can be applied to improve the implant local response to contamination. Considering the promising combination of nanostructured surfaces with antibacterial materials, this critical review describes a variety of antibacterial effects attributed to specific metals, ions and their combinations. Also, it explains the TiO_2_ nanotubes (TNTs) surface creation, in which the possibility of aggregation of an active drug delivery system is applicable. Also, we discuss the pertinent literature related to the state of the art of drug incorporation of NPs with antibacterial properties inside TNTs, along with the promising future perspectives of in situ drug delivery systems aggregated to biomedical implants.

## Introduction

Biomedical implants have their most critical moment of integration to living tissues when their biomaterial first contacts the human cells and local microorganisms (Yue et al. 2015). Researchers and clinicians expect the best biomaterial performance during this first contact, and enhance it by changing surface properties and morphology to boost the speed and quality of the healing process (Kunrath and Hubler 2018). However, implant surface contamination by bacterial agents might pose a significant threat, jeopardizing implant healing and/or affecting significantly its long-term survival (Sridhar et al. 2015; Raphel et al. 2016).

Nanoscale changes in implant surfaces have been the focus of several currently reported studies. The possibilities involved in surface nanotexturization are diverse, and studies show that these surfaces might offer substantial advantages regarding bacterial adhesion, bacterial proliferation and bone healing (Coelho et al. 2014; Truong et al. 2015; Kunrath and Hubler 2018). Following this idea, a nanotexturization method using electrochemical anodizing allows the formation of TiO_2_ nanotubes (TNTs), which substantially alter the physico-chemical properties of the implant surface making it friendlier to human bone cells and promoting potential antibacterial properties (Huang et al. 2017; Zhukova et al. 2017).

TNTs allow properties changes in terms of surface roughness, energy, wettability, tube diameter and possibly its greatest advantage, the alternative of incorporation of components as antibiotic drugs and other materials with similar potential, along with organic chemicals such as specific proteins, cytokines and growth factors (Hemeg 2017; Awad et al. 2017). The incorporating of such characteristics might allow the concept of a drug delivery system incorporated to those biomedical implants, as numerous drugs might be integrated to the tubes and released in situ through the activation of specific trigger mechanisms, which might be activated for local infection control as needed. (Wang et al. 2017a, b, c; Kunrath et al. 2018).

Infections contracted during the surgical act and/or after the healing process represent one of the potential risks to induce failure of a biomedical implant. Therefore, studies have been proposed aiming the development of drug incorporation systems like nanomaterials with antimicrobial properties to minimize those associated risks (Truong et al. 2015). Many ions and metal particles have been described to present antibacterial properties, which can be used in TNTs (Hemeg 2017). Nonetheless, biocompatibility and citotoxicity are features to be carefully evaluated before their safe indication as applicable tools in vivo (Lewinski et al. 2008). Innovative studies are nowadays testing these possibilities regarding drug incorporation with nanomaterials, evaluating their responses against bacterial colonization and cell adhesion (Liu et al. 2016; Yao et al. 2018).

In addition, TNTs surfaces allow the development of resorbable coatings over their tubes, which could maintain these added drugs viable, including antibiotics and other potential antibacterial agents, as well as to promote their slow release to the surrounding tissues if needed (Chen et al. 2013a, b; Kumeria et al. 2015). Preliminary studies with biodegradable coatings show promising results of drug release systems up to 30 days after surgical implantation. This might provide the basis for the development of a long-term release system activated by a specific mechanism without favoring bacterial drug resistance (Gulati et al. 2012).

The present critical review aims to describe the currently available nanoscale metallic materials with potential antibacterial properties already reported in the literature, detailing the TNTs surface construction process for biomedical implants along with their specific properties that might influence the adhesion and proliferation of microorganisms. In order to do that, the present review illustrates the status quo of nanoparticles/drugs with antibacterial properties incorporated in the TNTs and their preliminary reported test results, including a summary of coating possibilities and alternatives of late drug release mechanisms.

### Nanostructures and materials vs. antibacterial properties

Several materials and natural structures are studied searching for similarities in their intrinsic natural properties that could be adapted and used as biomaterials (Hasan et al. 2013). Some natural tissue surfaces have the ability to inhibit bacterial adhesion and proliferation, as well as alter their surface wettability. Natural-occurring examples are found in plant leaves, skin of aquatic animals, insect wings, among other structures that present antibacterial properties or some effect that might hinders bacterial proliferation (Bhadra et al. 2015; Jaggessar et al. 2017).

Currently, implants are produced using synthetic and non-synthetic materials with nanosurfaces, many of them through lithography, laser, or receiving chemical treatments (Zhang et al. 2006; Penha et al. 2018). Due to nanotopographical characteristics, the promotion of cellular interaction is substantial, since the extracellular matrix contact with the prepared surface occurs in nanoscale (Yim et al. 2010). Applied biomaterials are expected to provide faster tissue healing where they are inserted without any generated proinflammatory reactions, including degradation in vivo or corrosion. In addition, they might present properties to mitigate or even inhibit bacterial contamination (Bettinger et al. 2009; Neoh et al. 2012).

In this context, many metals have been studied in their natural atomic size, as well as fragmented to a condition of nanoparticles (NP), presenting potential properties (Kim and An 2012; Hemeg 2017). As already known, metals such as Zinc, Silver, Gold, Cobalt, Nickel and Lead have natural antibacterial characteristics, since their interaction with bacteria usually generates cellular structural damages, and complications in bacterial adhesion and proliferation (Dizaj et al. 2014; Hemeg 2017).

Therefore, many of these materials, when brought to NPs scale, have their antibacterial properties intensified, since they are able to interact more rapidly and effectively at nanoscale level (Hemeg 2017; Lee et al. 2018). Their main factor related to antibacterial action is associated with the production of hydrogen peroxide, superoxide anions and free hydroxyl radicals that induce countless damages to bacteria, such as membrane disruption, oxidative stress, changes in DNA, interference in the biofilm formation, among others (Oktar et al. 2015; Durán et al. 2016). The electrostatic interaction between the NPs and microorganisms affects its toxicity. Some NPs when connecting to bacterial cell surface release metal ions that bind and disrupt the cell membrane. Likewise, the metal adsorption results in oxidative stress due to ROS generation, which can lead to cell membrane damage. Furthermore, penetration of metal ions across the bacterial membrane results in damages to DNA by interaction with nitrogenous bases, which generate inhibition of DNA replication or lead to DNA degradation. In addition, metal ions can bind to ribosome subunits, inhibiting the protein synthesis (Hajipour et al. 2012; Hemeg 2017; Lee et al. 2018). Another important action of NPs is their interaction with biofilm. Biofilm is formed by microorganisms involved in a self-produced polymeric matrix that mediate the adhesion to a surface, acting as a physical barrier against antimicrobials (Kumar et al. 2017). However, NPs such as TiO_2_, Ag, ZnO, Cds, MgF_2_, Bi and YF_3_ can inhibit bacterial biofilm formation or disrupt formed biofilms (Hemeg 2017).

To clarify the antibacterial properties of metals, Table 1 shows the results of studies on different bacterial species and the generated effects in terms of their function and tissues, focusing mainly on the use of NPs derived from base metals. NPs are able to inhibit the adhesion, proliferation and to cause damage both in gram-positive and gram-negative bacteria, but some studies have demonstrated that NPs derived from NiO, Cu, Al_2_O_3_, SiO_2_ and Fe_2_O_3_ should be more effective against gram-positive bacterial strains (Baek and An 2011; Ruparelia et al. 2008; Jiang et al. 2009; Azam et al. 2012), suggesting a greater vulnerability of gram-positive bacteria to NPs. However, other studies indicated that Au and ZnO NPs are most effective against gram-negative pathogens, due to the higher thickness of the peptidoglycan layer in gram-positive bacteria (Shamaila et al. 2016; Sinha et al. 2011). In addition, Ruparelia et al. (2008) demonstrated that silver NPs should be more effective against *E*. *coli* (gram-negative bacteria) than *B*. *subtilis* (gram-positive bacteria). Conversely, Yoon et al. (2007) found a better efficiency of Ag NPs against *B*. *subtilis* than *E*. *coli*. Therefore, it should be considered that there are many variables among the studies, such as NP size and concentration, and the method used to evaluate the antimicrobial activity.

**Table 1:** Nanoparticles with antibacterial activity and the effects generated on bacterial species

Materials or NPs	Target bacteria	Effects	References
TiO_2_	*Pseudomonas aeruginosa*, *Escherichia coli*, *Staphylococcus aureus*, *Bacteroides fragilis*, *Enterococcus hirae*, *Salmonella* Typhimurium, *Streptococcus mutans*	Bactericidal effect after photoactivation of TiO_2_; membrane disruption; peroxidation of the polyunsaturated phospholipid component of the lipid membrane; loss of respiratory activity; induction of ROS generation; DNA damage and cell death	Hajipour et al. (2012), Tsuang et al. (2008), Kumar et al. (2011a, b), Seil and Webster (2012), Cui et al. (2012a, b), Ali et al. (2016)
Ag	*Bacillus subtilis*, *Enterobacter* sp., *Marinobacter* sp., *Pseudomonas putida, E. coli*, *Vibrio cholerae*, *Salmonella* Typhi, *P. aeruginosa, Acinetobacter baumannii*, *Clostridium diphtheriae*, *S. aureus*, *Streptococcus pyogenes*, *Staphylococcus epidermidis*, *Enterococcus faecalis*, *Klebsiella pneumoniae*, *Listeria monocytogenes*, *Proteus mirabilis*, *Micrococcus luteus*, *S. mutans*	Reduction in bacterial growth and viability; electrostatic interactions with bacterial membrane; action on the cellular permeability and respiration; interaction with organelles and biomolecules; ROS generation; modulation of cell signaling; DNA damage	Sinha et al. (2011), Jiang et al. (2009), Kumar et al. (2011a, b), Lee et al. (2018), Hajipour et al. (2012), Gajjar et al. (2009), Seil and Webster (2012), Morones et al. (2005), Dakal et al. (2016), Rai et al. (2012), Franci et al. (2015), Nour El Din et al. (2016), Ruparelia et al. (2008), Hemeg (2017), Yoon et al. (2007)
Au	*S*. *aureus*, *E*. *coli*, *B. subtilis*, *K*. *pneumoniae*, *P*. *aeruginosa*	Inhibition of bacterial growth; change in the membrane potential, reduction of respiratory and ATPase activities, and inhibition of subunit of the ribosome preventing tRNA binding; optical properties; bacterial membrane disruption	Shamaila et al. (2016), Cui et al. (2012a, b), Huo et al. (2016), Hemeg (2017)
ZnO	Halophilic *bacterium*, *Marinobacter* sp., *B. subtilis, Enterobacter* sp., *E. coli*, *Pseudomonas fluorescens, S. aureus, P. aeruginosa, S.* typhimurium*, P. putida*	Inhibition of bacterial growth; change in cell morphology and reduction in cell size; cell membrane disruption and accumulation of nanoparticles in the cytoplasm; electrostatic interactions with bacterial membrane; induction of ROS production, DNA damage and cell death	Hajipour et al. (2012), Sinha et al. (2011), Jiang et al. (2009), Kumar et al. (2011a, b), Azam et al. (2012), Gajjar et al. (2009), Feris et al. (2010), Baek and An (2011), Sirelkhatim et al. (2015), Esparza-Gonzalez et al. (2016), Seil and Webster (2012)
Cu	*B. subtilis*, *E. coli*, *P. aeruginosa*, *S. aureus*, *P. putida*, *M*. *luteus*, *K*. *pneumoniae*	Reduction of bacterial growth; dissipation of cell membrane potential; ROS generation; lipid peroxidation; protein oxidation and DNA degradation	Azam et al. (2012), Lee et al. (2018), Gajjar et al. (2009), Chatterjee et al. (2014), Yoon et al. (2007), Ramyadevi et al. (2012), Bogdanovic et al. (2014), Ruparelia et al. (2008), Baek and An (2011)
Se	*S. aureus*, *E. coli*	Reduction of bacterial growth	Guisbiers et al. (2016)
SiO_2_	*E*. *coli*, *B*. *subtilis*, *P*. *fluorescens*	Membrane disruption; reduction of bacterial growth	Lee et al. (2018), Seil and Webster (2012), Jiang et al. (2009)
NiO	*Streptococcus pneumoniae*, *E. coli*, *B. subtilis*, *S. aureus*, *P. aeruginosa*	Increase of bacterial wall permeability; inhibition of microbial growth associated with intrinsic toxic properties of metal	Khashan et al. (2016), Baek and An (2011)
Al_2_O_3_	*E*. *coli*, *B*. *subtilis*, *P*. *fluorescens*	Reduction of bacterial growth by particle penetration and cell wall damage	Lee et al. (2018), Seil and Webster (2012), Hajipour et al. (2012), Jiang et al. (2009), Simon-Deckers et al. (2009), Ansari et al. (2014)
Fe_2_O_3_	*E*. *coli*, *B*. *subtilis*, *P*. *aeruginosa, S*. *aureus, S*. *epidermidis*	Inhibition of bacterial growth	Azam et al. (2012), Taylor and Webster (2009), Seil and Webster (2012)
Y_2_O_3_	*E*. *coli*, *S*. *aureus*, *P*. *aeruginosa, Serratia marcescens*	Inhibition of bacterial growth	Kannan and Sundrarajan (2015), Lee et al. (2018)
YF_3_	*E*. *coli*, *S. aureus*	Reduction of bacterial colonization on YF_3_ coated-surface and antibiofilm activities	Lellouche et al. (2012a, b), Hemeg (2017)
CdS	*E*. *coli*	Antibiofilm activity	Dhanabalan and Gurunathan (2015), Hemeg (2017)
MgF_2_	*E*. *coli*, *S*. *aureus*	Inhibition of biofilm formation; ROS generation, lipid peroxidation and penetration of cell envelope	Lellouche et al. (2012a, b), Hemeg (2017)
Bi	*S*. *mutans*	Reduction of bacterial growth; inhibition of biofilm formation	Hernandez-Delgadillo et al. (2012), Hemeg (2017)

*NPs* nanoparticles, *ROS* reactive oxygen species

### TiO_2_ nanotubes (TNTs)

#### Anodization process

Physical and chemical treatments for Ti have been proposed in order to obtain surfaces with better biocompatibility. Among these techniques, anodization has been recognized for improving wear and corrosion resistance, as well as increasing TiO_2_ surface roughness and surface porosity, with varying thicknesses (Liu et al. 2012). In addition, it is considered a methodology that easily reproduces results, being accessible and inexpensive, and also able to facilitate researchers to test their properties with different scientific bases (Awad et al. 2017).

Surface modification has been reported to be an effective tool to promote the integration between bone and biomaterials (Minagar et al. 2012). Anodizing is an effective electrochemical method that has been used successfully in orthopedic implants surface treatment (Liu et al. 2004). It can be performed in an electrochemical cell with two electrodes (usually titanium anode, platinum or titanium cathode). The oxidation and reduction reactions occur at the anode when a current or constant voltage is applied, thus establishing an electric field that guides ion diffusion present in the electrolyte, leading to oxide film formation on the surface of the anode.

The chemical and structural properties of anode oxides vary according to the different solutions used as electrolytes. The anodization electrochemical parameters significantly affect film growth behavior and properties. Such features include the type of solution used as electrolyte, reagent concentration, temperature, electrical parameters, anodizing time and solution stirring speed (Liu et al. 2004; Cui et al. 2009; Liu et al. 2012). The anode potential and the electric current can alter the anion transfer process during anodization, as well as determining thickness, surface morphology and microstructure of the anodic coatings (Awad et al. 2017; Kunrath et al. 2018).

**Fig. 1:**
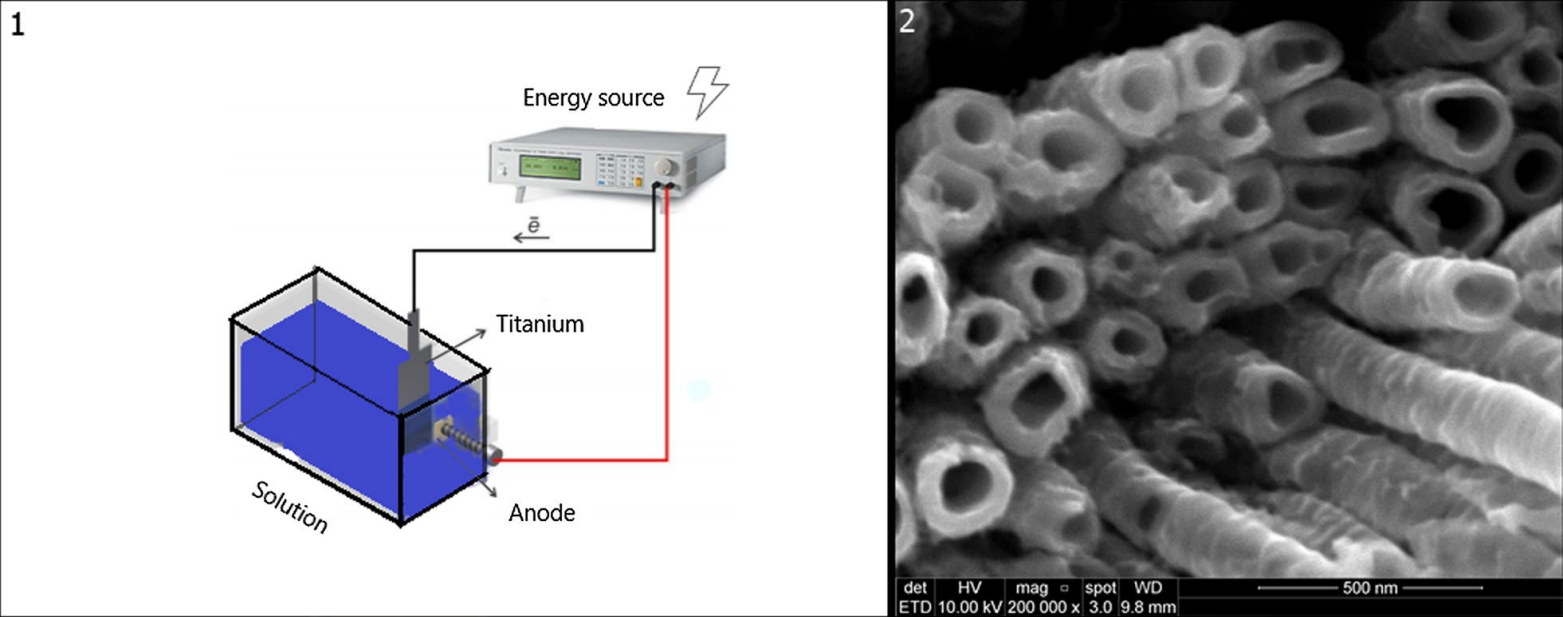
Anodization process explanatory scheme (**1**). TiO_2_ nanotubes made through anodization (**2**)

From the control of this entire process and selection of the correct protocols, TNTs can be developed with dimensions and properties suitable for biomedical implants as shown in Fig. 1.

#### TiO_2_ nanotubes properties and advantages

**Fig. 2:**
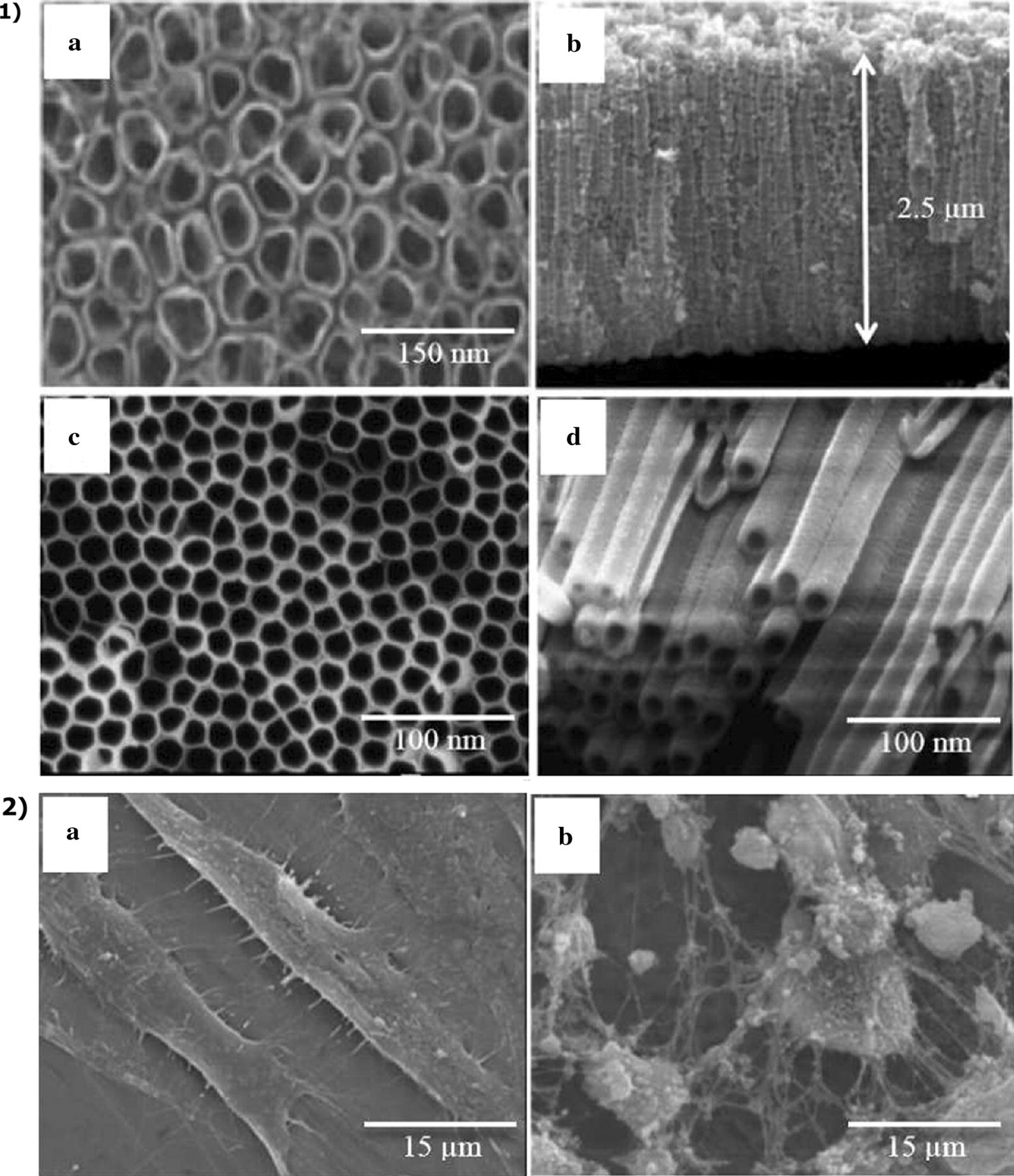
Alteration in nanotube diameter and morphology only by variation of the solution used and voltage parameters. 1 M solution (NH_4_) 2SO_4_ + NH_4_ F at 20 V (**1a**, **b**) and NH_4_F + H_2_O + ethylene glycol at 60 V (**1c**, **d**). Adhesion of osteoblastic cells on machined surface (**2a**) and surface of TiO_2_ (**2b**) nanotubes [Reprinted and adapted with permission from Elsevier, (Awad et al. 2017)]

Small changes in the anodization process directly influence the properties of TNTs, such as surface roughness, wettability, cellular interaction, drug loading capacity and chemical physical structure, as can be seen in Fig. 2 (Awad et al. 2017). Nanotubes surfaces with greater roughness and very low wettability angles have been described to allow expressive responses in adhesion and proliferation of mesenchymal or bone cells (Vasilev et al. 2010; Yu et al. 2018). On the other hand, some investigations have shown TNTs surfaces with hydrophobic properties might positively influence bacterial adhesion (Zhang et al. 2013).

Important biological properties associated with implant surfaces are hugely influenced by changes in tube morphological characteristics. A great number of studies applying cell culture techniques on TNTs presented better cell adhesion and proliferation on nanotubes of 70 nm in diameter (Awad et al. 2017; Yu et al. 2018), although expressive results were also reported when applying diameters ranging from 30 to 100 nm (Yu et al. 2018).

Antibacterial properties of TNT surfaces were evaluated in comparison with bacterial responses to microtextured surfaces (Miao et al. 2017). As most bacteria and/or viruses have their size compatible to a micrometric scale, the nanoscale topography presented in TNTs seemed to reject or even hinder their growth in culture (Jaggessar et al. 2017; Miao et al. 2017; Lee et al. 2018) Another observed advantage is that TNTs chemical–physical structure presents great resistance to corrosion and degradation in vivo, showing promising results considering application in biomedical implants (Alves et al. 2017).

Despite these numerous advantages, the greatest differential of the TNTs technology relies on the possibility of drug or NPs incorporation in the tubes. As described by some authors, this might allow even the creation of a “smart” drug transport system, allowing that a drug could be stored and late released in situ in an on-demand basis, not possible in any other surface treatment currently used in biomedical implants (Wang et al. 2017a, b, c; Awad et al. 2017).

### Wettability and bacterial adhesion

One of the most influential properties regarding cell or bacterial adhesion to metallic implants is surface wettability. This property can be modified with consequent treatments of the nanotubes applying thermal, chemical, photofunctionalization, and coating treatments (Oliveira et al. 2017; Hasan et al. 2013; Lai et al. 2016).

**Fig. 3:**
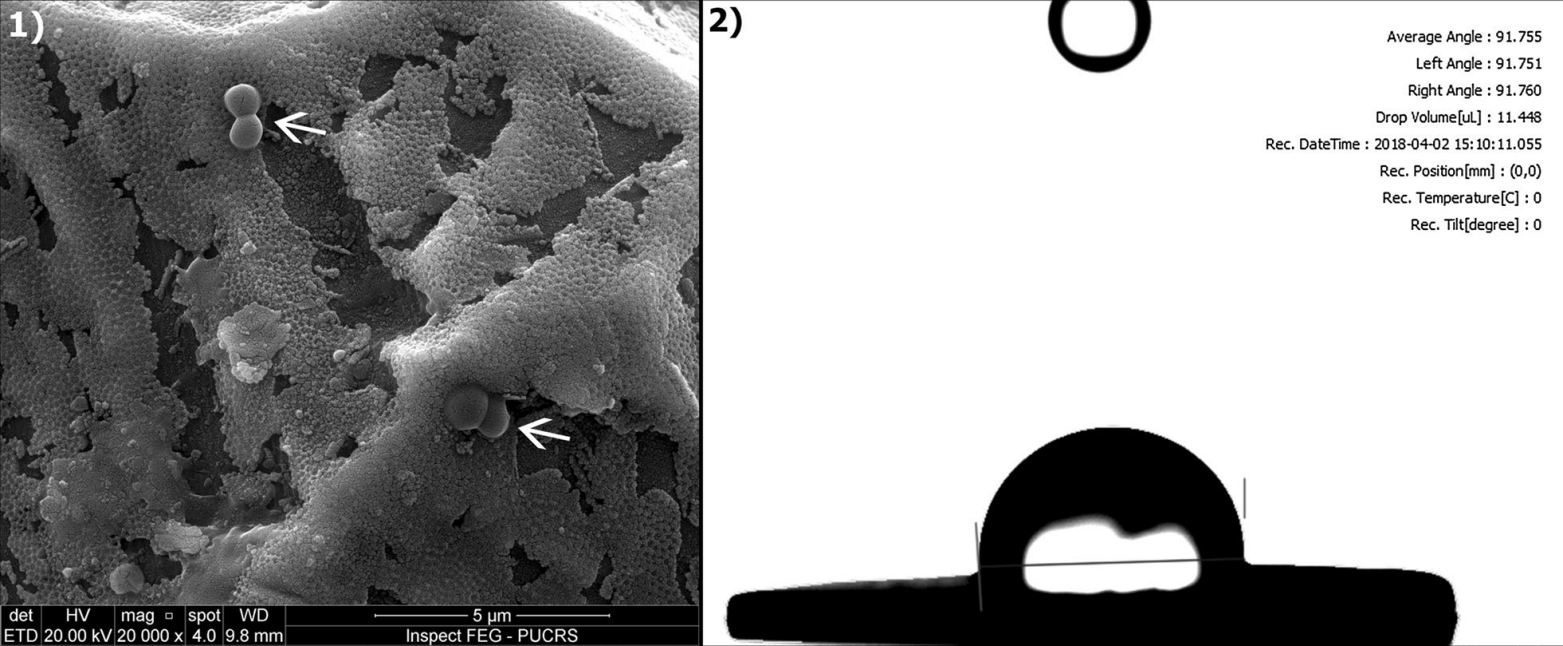
SEM image showing (white arrows) a nanotube surface after adhesion assay with *Staphylococcus epidermidis* and fixation (**1**), and its previous characterization regarding wettability indicating hydrophobicity (**2**)

Studies on hydrophobic surfaces showed important bacterial anti-adhesion properties and promotion of bone cells growth (Lai et al. 2016; Fadeeva et al. 2011; Gittens et al. 2014). These results might be extrapolated for nanotubes surface development aiming the promotion of bacterial repulsion, combined with high biocompatibility as depicted in Fig. 3 (Xu and Siedlecki 2014; Mei et al. 2014).

Synthetic and/or non-synthetic coatings, which are used to keep added drugs in the internal parts of the nanotubes, or to protect the TNT system, usually present hydrophobic properties (Oliveira et al. 2017; Kumeria et al. 2015). The adsorption of bacterial extracellular matrix to these surfaces might be impaired, turning bacterial local proliferation and consequently biofilm formation unlikely (Xu and Siedlecki 2014).

However, other studies characterized hydrophilic surfaces as presenting better adhesion and proliferation conditions for bone-like and undifferentiated mesenchymal cells, as well as pre-osteoblasts (Lotz et al. 2018; Gittens et al. 2014). This may be a counterpoint compared to hydrophobic surfaces. Nonetheless, as described above, other studies showed better antibacterial performance on hydrophobic surfaces, and this surface wettability vs. bacterial/cell relation is still not fully clarified.

#### Drug delivery system

Traditional therapies to prevent or treat infection/inflammation in surrounding tissues of biomedical implants are usually administered systemically. However, systemic drug administration does not target a specific site and may not interact directly at the desired site. The possibility of having a localized and selective drug delivery system for biomedical implants acting as an aid or even replace a systemic drug administration becomes extremely promising (Lyndon et al. 2014; Hemeg 2017).

It has been stated that regarding implant surgery, osteoblasts and bacteria establish a marked competition for implant surface adhesion, and the alternative of loading TNTs with antibacterial drugs might be of great advantage (Miao et al. 2017). Other studies have also suggested that the successful loading of TNTs with antimicrobial peptides for later local release might positively contribute to raise levels of infection prevention, or the combination of an initial rapid release with an extended slow and gradual release (Li et al. 2017).

Investigations suggested that some bacteria such as *Staphylococcus aureus*, *Staphylococcus epidermidis* and *Escherichia coli* are often present in implant postoperative infections and negatively affect the healing process of bone. In order to combat this local cellular competition more emphatically, the alternative of improving surface properties with antibacterial characteristics becomes crucial (Anselme et al. 2010; Widaa et al. 2012). Alternatively to antibacterial drugs, application of specific metal particles might also be considered due to their specific bacteriostatic and bactericidal activities. Metallic elements such as ZnO-NPs presented excellent ability to control *S. aureus* when applied to TNTs (Yao et al. 2018). In this context, an investigation on loading of ZnO nanoparticles of irregular and regular shapes to the implant surface and later testing it against bacterial proliferation as well as macrophages viability was conducted. The results suggested a ZnO capacity to inhibit bacterial growth and macrophage adhesion, which might ultimately result in lower levels of local inflammation around implants (Yao et al. 2018).

Silver nanoparticles loaded onto TNTs showed a significant antibacterial effect against *E. coli* at both contact kill analysis and agent release killing effect, presenting promising results (Chen et al. 2013a, b). Copper nanocubes (20 nm) were also loaded onto TNTs and showed effective antibacterial action against *S. aureus* and *E. coli*, causing eradication of these bacteria in laboratorial cultures (Rosenbaum et al. 2017).

In addition to NPs derived from metals, studies showed also the possibility of TNTs loading with commonly used systemic antibiotics, especially those targeting bacterial cell wall and protein synthesis. Investigations have presented in vitro and in vivo results involving TNTs loaded with vancomycin (Zhang et al. 2013). Preliminary data suggested better in vitro antibacterial performance against *S. aureus* in the groups with nanotubes loaded with antibiotics compared with TNTs-only control group (Zhang et al. 2013), suggesting that the application of antibiotics in situ might be a viable alternative appropriate to this technology.

Different outcomes of antibiotic loaded TNTs where verified, and most often antibiotics might generate particularly interesting results. A pioneer study in TNTs antibiotic loading used doses of gentamicin against *S. epidermidis* in vitro (Popat et al. 2007). Their results showed complete TNTs filling, as well as reduction in bacterial surface adhesion and an increase in the proliferation rate of pre-osteoblasts where the antibiotic was used, revealing a double positive result without interfering with implant biocompatibility (Popat et al. 2007).

The most recent studies employing incorporation of drugs, specific agents and NPs as antibacterial agents into TNTs is summarized in Table 2, where their promising results are described, revealing also the “state of the art” of this technology.

**Table 2:** Antibacterial drugs and NPs loaded in TiO_2_ nanotubes

NPs/drugs	Target bacteria	Results	References
Antimicrobial peptides	*Staphylococcus aureus, Pseudomonas aeruginosa, Fusobacterium nucleatum, Porphyromonas gingivalis*	Eradication of bacterial growth in vitro; killing of 99.9% of the bacteria; reduction of bacterial adhesion; activity against planktonic and adhered bacteria; absence of cytotoxicity to osteoblasts and cytocompatibility	Kazemzadeh-Narbat et al. (2013), Ma et al. (2012), Zhang et al. (2017), Li et al. (2017)
Gentamicin	*Staphylococcus epidermidis*	Significant reduction of bacterial adhesion; drug release from nanotubes grown on the ultrafine-grained (UFG) titanium is slower than grown on the coarse-grained (CG) titanium	Popat et al. (2007), Nemati and Hadjizadeh (2017)
Gentamicin/chitosan	*S*. *aureus*	Inhibition of bacterial adherence, enhance of cell viability and maintenance of drug release	Feng et al. (2016)
Vancomycin	*S. aureus*	Biocompatibility and reduction of bacterial adhesion; long release time and bacterial inhibition	Zhang et al. (2013), Ionita et al. (2017)
Penicillin	ND	Biocompatibility and decrease of bacterial cell functions	Yao and Webster (2009)
Zn	*S*. *aureus, Streptococcus mutans*	Inhibition of bacterial proliferation and viability; morphological change, inhibition of proliferation and adhesion of macrophages	Yao et al. (2018), Roguska et al. (2018)
Sr/Ag_2_O	*S*. *aureus*	Antibacterial effect, osteogenic and angiogenic activities	Chen et al. (2017)
Sr/Ag	Methicillin-resistant *S. aureus* (MRSA) and methicillin-sensitive *S. aureus*, *Escherichia coli*	Antibacterial and anti-adherent properties; absence of cytotoxicity	Cheng et al. (2016)
Cu	*S*. *aureus*, *E*. *coli*	Reduction of bacterial adhesion	Rosenbaum et al. (2017)
Au	*S*. *aureus*, *E*. *coli*	Antibacterial effects against the bacteria for a total time of 21 days; cytocompatibility with osteoblasts; alteration of bacterial membrane; moderated antibacterial effect	Wang et al. (2017a, b, c), Wang et al. (2016), Li et al. (2014), Yang et al. (2016)
Carbon	*S*. *aureus*, *E*. *coli*	Increase of antibacterial effects after an electric induction; cytocompatibility with osteoblasts	Wang et al. (2018)
Ag	*S*. *aureus*, *E*. *coli, S*. *mutans,* ND	Bacterial killing and inhibition of bacterial adhesion; kill all bacteria suspension at the first days and have the ability to prevent the bacterial adhesion in the next days; effectively kill bacteria even after immersion for 28 days; absence of cytotoxicity; growth inhibition of oral pathogens; biocompatibility in vivo and in vitro; reduction of inflammatory responses in vivo; adhesion and proliferation of fibroblasts	Zhao et al. (2011), Gao et al. (2014), Roguska et al. (2018), Mei et al. (2014), Uhm et al. (2014), Piszczek et al. (2017), Esfandiari et al. (2014)
Ca/P/Ag	*S*. *aureus*	Inhibition of bacterial growth; enhancing of adhesion and spreading of osteoblasts	(Li et al. 2015)

*NPs* nanoparticles, *ND* not determined

#### Biomolecules and NPs immobilization through coatings

As the demand of active functionalization of different biomedical implant surfaces rises, the need to investigate how to properly seize/immobilize different types of drugs or NPs on the implant surface escalates. Thus, a series of investigations suggesting the use of ceramic, organic, or inorganic materials as implant coatings are being conducted with the objective of creating a protective stable but resorbable layer that can gradually release the drug once needed (Civantos et al. 2017).

**Fig. 4:**
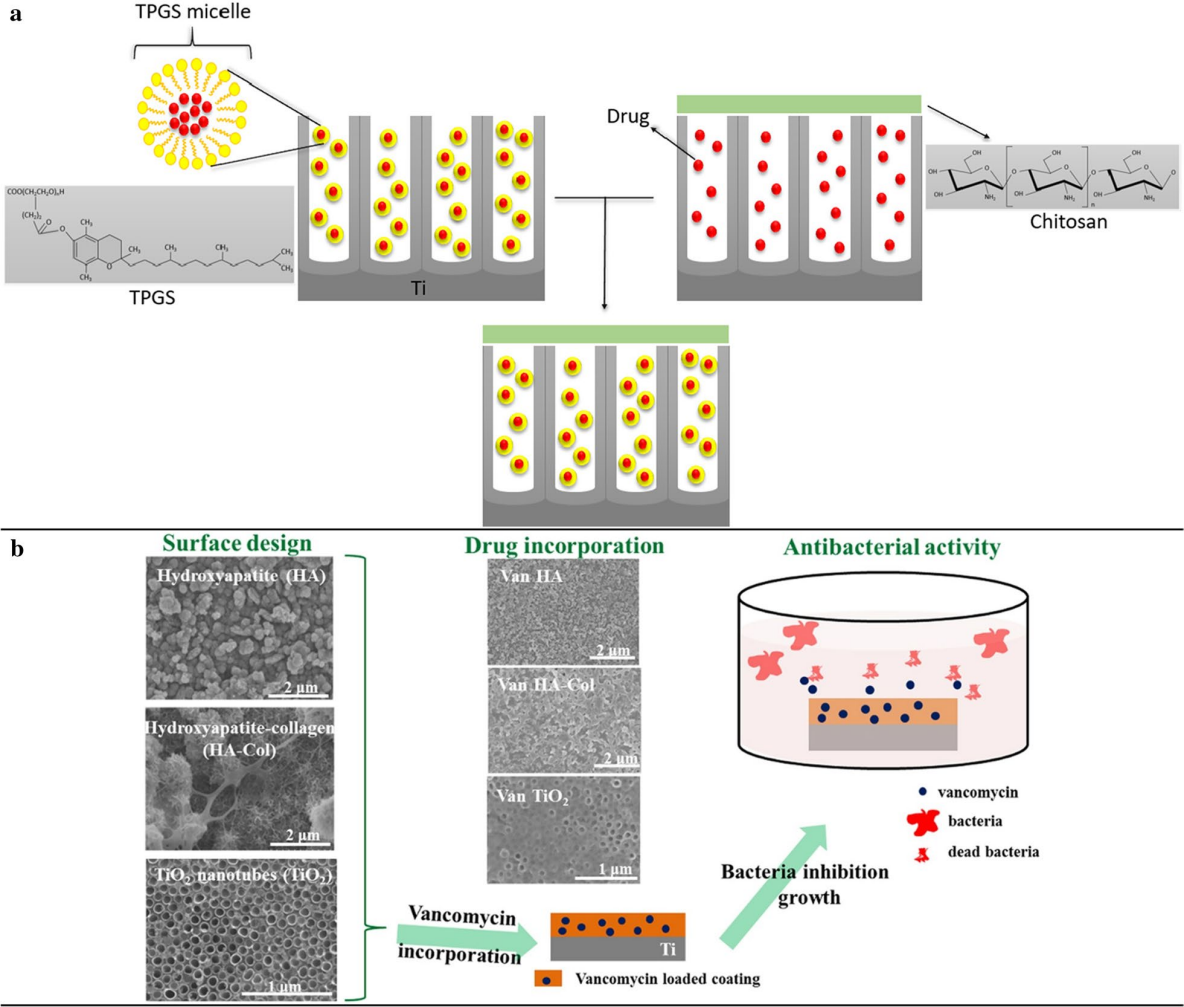
Schematics showing how coated TNTs may present stabilized drugs in their tubes and how some materials are applied to these coatings (**a**), reprinted with permission from Elsevier, (Oliveira et al. 2017). Scheme showing how the release of drugs influences the behavior of bacteria (**b**), reprinted with permission from Elsevier, (Ionita et al. 2017)

The TNTs surfaces offer the possibility of chemical–physical coating adhesion on their surface along with drug incorporation to their tubes. When evaluated in vitro, biodegradable coatings showed excellent results on titanium surfaces, positively influencing their biocompatibility with expressive results of bone cell proliferation and adhesion (Goodman et al. 2013; Oliveira et al. 2017). In addition, progressive coating degradation has been characterized as a viable event that may allow the release of drugs or NPs aiming to potentiate bone healing or even prevent possible microbial infections (Oliveira et al. 2017). Figure 4 shows a schematic of how TNTs with coatings stabilize the drugs in their tubes and materials, which can be used to make these coatings.

Furthermore, some studies have even extrapolated the incorporation of drugs or growth factors (GFs) not only to their TNTs but into their coatings as well. Chitosan coatings incorporated with GFs presented better results in terms of cell adhesion, bone cell proliferation and increase in the expression of bone formation markers in vitro (Abarrategi et al. 2007). Also, Chitosan coatings were tested for antibiotic incorporation in vivo presenting promising results as well (Jennings et al. 2015; Lai et al. 2017).

The combination of loaded TNTs with a drug-incorporated surface coating might represent an attractive possibility for in loco treatment of a failing and/or compromised implanted device. However, studies investigating this possibility are scarce and, when available, still at early stages, and so far there is no single definition in terms of the best surface coating material or the ideal drug to be implemented for these purposes in vivo.

One attempt to investigate the possibilities involving surface coating association with TNTs drug delivery system was related to late local antibiotic-release activation (Wang et al. 2017a, b, c). In this investigation, nanotubes were firstly loaded with antibiotics and then sealed with coordination polymers through a metal ion bond, generating a hybrid system with potential antibacterial properties. Reported in vitro observations revealed that the antibiotic agent was kept inside the tubes until the surrounding environment had its pH altered. As the inflammatory process elicited by bacterial infection has the potential to change the local pH, this in vitro drug release system was activated, suggesting a possibility for long-term local drug maintenance prior to its late release (Wang et al. 2017a, b, c).

In conclusion, the present overview critically analyzed the significant diversity of drugs, metals and NPs presenting antibacterial properties and their functionalization on TNTs surfaces, depicting a wide range of these potential metals and drugs used in biomedical implants and related advanced research regarding their biocompatibility and potential antibacterial effects.

TiO_2_ nanotubes surfaces show a large energy area for immobilization and subsequent functionalization of nanoparticles, preserving its biocompatibility and mechanical stability. Many strategies to incorporate NPs and drugs to implant surfaces have been presenting promising preliminary results in combating bacteria that cause infections in the human, and especially where biomedical implants may be placed.

It has been suggested by some investigations that the drug delivery system applied to TNTs might be successful and has promising early results. Even though a large part of these reported studies represent in vitro evidence, the pathway for clinical trials involving concepts on the application of drug delivery TNTs has already been suggested, followed by more extensive cytotoxicity tests and in vivo observations to firstly ensure their biologic safety.

The possibility of decreasing the application of systemic antibiotics usually associated with side effects to patients for an in situ usage seems to be one of these drug-loaded TNTs most advantageous properties, since their association with surface coatings may keep a drug and/or NP active for a long-term gradual release once needed. Further investigation in vivo should be performed to establish the proper advantages and efficacy linked to the in situ application of antibiotics in biomedical implants compared to systemic drug administration.

Finally, the use of functionalized TNTs aiming a localized release of antibiotic drugs and bactericide NPs has presented promising early results and should be further explored aiming future medical applications. The possibilities related to local infection control may positively influence the outcome of a major biomedical problem related to implant loss and/or disfunction. With established protocols regarding adequate drug dose and production optimization, this technology can play an important role into further increase the clinical success of biomedical implants in the near future.

## Abbreviations

DNA: deoxyribonucleic acid
GFs: growth factors
NPs: nanoparticles
ROS: reactive oxygen species
TNTs: TiO_2_ nanotubes
